# Risk Prediction of Pancreatic Cancer in Patients With Abnormal Morphologic Findings Related to Chronic Pancreatitis: A Machine Learning Approach

**DOI:** 10.1016/j.gastha.2022.06.008

**Published:** 2022-06-17

**Authors:** Wansu Chen, Qiaoling Chen, Rex A. Parker, Yichen Zhou, Eva Lustigova, Bechien U. Wu

**Affiliations:** 1 Department of Research and Evaluation, Kaiser Permanente Southern California Research and Evaluation, Pasadena, California; 2 Department of Radiology, Los Angeles Medical Center, Southern California Permanente Medical Group, Los Angeles, California; 3 Department of Gastroenterology, Center for Pancreatic Care, Los Angeles Medical Center, Southern California Permanente Medical Group, Los Angeles, California

**Keywords:** Pancreatic Cancer, Machine Learning, Risk Prediction, Chronic Pancreatitis, Imaging Features

## Abstract

**Background and Aims:**

A significant factor contributing to poor survival in pancreatic cancer is the often late stage at diagnosis. We sought to develop and validate a risk prediction model to facilitate the distinction between chronic pancreatitis–related vs potential early pancreatic ductal adenocarcinoma (PDAC)-associated changes on pancreatic imaging.

**Methods:**

In this retrospective cohort study, patients aged 18–84 years whose abdominal computed tomography/magnetic resonance imaging reports indicated duct dilatation, atrophy, calcification, cyst, or pseudocyst between January 2008 and November 2019 were identified. The outcome of interest is PDAC in 3 years. More than 100 potential predictors were extracted. Random survival forests approach was used to develop and validate risk models. Multivariable Cox proportional hazard model was applied to estimate the effect of the covariates on the risk of PDAC.

**Results:**

The cohort consisted of 46,041 (mean age 66.4 years). The 3-year incidence rate was 4.0 (95% confidence interval CI 3.6–4.4)/1000 person-years of follow-up. The final models containing age, weight change, duct dilatation, and either alkaline phosphatase or total bilirubin had good discrimination and calibration (c-indices 0.81). Patients with pancreas duct dilatation and at least another morphological feature in the absence of calcification had the highest risk (adjusted hazard ratio [aHR] = 14.15, 95% CI 8.7–22.6), followed by patients with calcification and duct dilatation (aHR = 7.28, 95% CI 4.09–12.96), and patients with duct dilation only (aHR = 6.22, 95% CI 3.86–10.03), compared with patients with calcifications alone as the reference group.

**Conclusion:**

The study characterized the risk of pancreatic cancer among patients with 5 abnormal morphologic findings based on radiology reports and demonstrated the ability of prediction algorithms to provide improved risk stratification of pancreatic cancer in these patients.

## Introduction

Pancreatic cancer is the third leading cause of cancer-related death in the United States among cancers that afflict both men and women.^1^ A significant factor contributing to poor 5-year survival in pancreatic cancer is the often late stage at diagnosis with more than 50% of patients harboring metastases at the time of presentation.^2^^,^^3^ However, the United States Preventative Services Task Force recently reissued guidance against widespread population-based screening citing several key gaps in current knowledge related to early detection.^4^ One of the key areas highlighted was the need for a better understanding of the natural history of precursor lesions in pancreatic cancer.

Chronic pancreatitis (CP) is a chronic inflammatory condition of the pancreas, which manifests clinically with chronic or recurrent episodes of abdominal pain, exocrine as well as endocrine insufficiency. Imaging plays a key role in the diagnosis of CP and frequently involves a multimodality approach including computed tomography (CT), typically with one or more contrast enhancement phases, magnetic resonance imaging (MRI) with or without magnetic resonance cholangiopancreatography, ultrasound, and endoscopic ultrasound all having a role.^5^^,^^6^ Imaging features include dystrophic calcifications, glandular atrophy, pancreatic duct dilatation, and cyst/pseudocyst development.

CP manifests histopathologically with loss of acinar cells, fibrosis, and chronic inflammatory cells. This dense stromal response resembles the desmoplasia often seen in the setting of pancreatic ductal adenocarcinoma (PDAC), which is thought to be mediated by activated myofibroblasts known as pancreatic stellate cells.^7^ CP is an established risk factor for pancreatic cancer with a recent meta-analysis by Kirkegard et al^8^ showed that 5 years after diagnosis patients with CP have a nearly 8-fold increased risk of pancreatic cancer. In addition, up to 5.5% of patients with suspected CP based on imaging are actually diagnosed with pancreatic cancer within 1 year of follow-up indicating underlying malignancy at the time of CP diagnosis.^9^

In this study, we focused on PDAC, a common type of pancreatic cancer. We hypothesized that some of the characteristic imaging features associated with CP may represent early changes associated with PDAC-related desmoplasia in the appropriate clinical setting. We therefore sought to perform a comprehensive assessment of the natural history of common imaging-related morphologic changes of the pancreas as well as develop and validate a risk prediction model to facilitate the distinction between CP-related vs potential early PDAC-associated changes on pancreatic imaging.

## Materials and Methods

### Study Design and Setting

This is a retrospective cohort study conducted based on multiethnic health plan enrollees of Kaiser Permanente Southern California (KPSC). KPSC is an integrated health care system that provides comprehensive health care services for more than 4.8 million enrollees across 15 medical centers and 250+ medical offices throughout the Southern California region. The study data elements were extracted from the Research Data Warehouse, which integrates the data from electronic health records (EHRs) and legacy systems dating back to the 1980s and is supplemented by radiology reports obtained from the data repository of the KPSC EHR. The race/ethnicity distribution, demographics, and socioeconomic status of KPSC health plan enrollees are comparable to those of the residents in the Southern California region.^10^ The study protocol was approved by KPSC’s Institutional Review Board.

### Cohort Identification and Follow-Up

Patients aged 18–84 years whose abdominal CT or MRI reports indicated duct dilatation, atrophy, calcification, cyst, or pseudocyst between January 1, 2008, and November 30, 2019, were identified using the natural language processing (NLP) algorithms previously reported.^11^ For patients who had more than one qualifying imaging study during the study period, one was randomly selected. The selection of a random image was performed to gain a representation of the extent of imaging-based pancreatic morphologic changes, given the cumulative nature of potential findings over time while mitigating potential immortal time bias. The randomly selected imaging procedure was referred to as the index scan, and the date of the index scan was referred to as the index date (t_0_). Exclusion criteria included reported mass in the pancreas >2 cm, history of pancreatic cancer, and enrollment in the health plan less than 12 continuous months before or 30 days after t_0_. The requirement of a continuous enrollment allowed adequate data to define study variables. For each patient in the cohort, follow-up started at t_0_ and ended with the earliest of the following events: disenrollment from the health plan, end of the study (December 31, 2019), reached the maximum length of follow-up (3 years), non-PDAC-related death, or PDAC diagnosis or death (outcome).

### Outcome Identification

The primary outcome was defined as the diagnosis of PDAC or death in the setting of pancreatic cancer in the 3 years after the index date. PDAC was captured from the KPSC Cancer Registry using the Tenth Revision of International Classification of Diseases, Clinical Modification (ICD-10-CM) code C25.x and histology codes listed in Supporting Document 1. The KPSC Cancer Registry is part of the Surveillance, Epidemiology, and End Results program. The pancreatic cancer deaths were derived from the linkage with the California State Death Master files and captured using ICD-10-CM codes C25.x. The utilization of the State files allowed the identification of pancreatic cancer cases that were not otherwise captured in the registry.^12^ However, the cases identified through the death files did not contain information on histology.

### Patient Demographic and Clinical Features at Baseline

A complete list of features included in the analyses is presented in Table 1. Diabetes was defined by International Classification of Diseases, Ninth Revision (ICD-9) or Tenth Revision (ICD-10) for diabetes (ICD-9: 250.x and ICD-10: E8-E13) or KPSC internal code (1200, 1201, 1202, 1203, 1204, 1839, 3141, 3186, 3639, 4124, or 5782), any prior glycated hemoglobin level >7.0%, or any dispensing record for insulin or an oral hypoglycemic medication (not including metformin; Table A1). Because all the laboratory values and weight measure and the changes of these values were not complete, “missRanger” was applied to impute the missing values if the frequency of missing for a feature was <60%.^13^ We used predictive mean matching method^14^ with k = 5. Laboratory measures with 60% or more missingness or change/change rate measures with 80% or more missingness were not included in the model development process. The missing values of weight-related features were handled in the same manner. Ten imputed data sets were generated.

**Table 1:** Characteristics of Study Subjects at Baseline (n = 46,041)

Patient characteristics	n (%) unless otherwise stated
Demographics and lifestyle characteristics	
Age, mean (SD)	66.4 (13.1)
Female	25,693 (55.8%)
Race/ethnicity	
Non-Hispanic White	23,558 (51.2%)
African American	5153 (11.2%)
Hispanic	12,538 (27.2%)
Asian and Pacific Islanders	4245 (9.2%)
Multiple/other/unknown	547 (1.2%)
Tobacco use	
Ever	22,260 (48.5%)
Never	23,679 (51.5%)
Unknown	102 (0.0%)
Diagnosis of alcohol abuse in the past year	3125 (6.8%)
Diagnosis of alcohol abuse any time in the past	5315 (11.5%)
Family history of pancreatic cancer	1452 (3.2%)
Weight, median (IQR), n = 45,291	168.2 (141.3, 200.0)
Weight group defined by body mass index (kg/m^2^)	
Underweight (<18.5)	1498 (3.3%)
Normal weight (18.5–24.9)	13,675 (29.7%)
Overweight (25–29.9)	15,242 (33.1%)
Obese (30+)	14,869 (32.3%)
Unknown	757 (1.6%)
Weight change in 1 y (kg), median (IQR), n = 38,591	−1.0 (−4.4, 1.5)
≤−6 kg	6752 (14.7%)
>−6 and ≤−4 kg	3722 (8.1%)
>−4 and ≤−2 kg	5451 (11.8%)
>−2 and <2 kg	14,367 (31.2%)
≥2 and <4 kg	3848 (8.4%)
≥4 kg	4451 (9.7%)
Unknown	7450 (16.2%)
Clinical characteristics	
Gallstone disorders	11,007 (23.9%)
Acute pancreatitis	5810 (12.6%)
Chronic pancreatitis	1881 (4.1%)
Benign pancreatic disease	3902 (8.5%)
Biliary tract disease	13,326 (28.9%)
Depression	16,742 (36.4%)
Deep vein thrombosis	2432 (5.3%)
Hereditary cancer syndromes	6647 (14.4%)
Active cancer (other than pancreatic cancer)	3487 (7.6%)
Peptic ulcer	5725 (12.4%)
Diabetes	
Within 6 mo	15,469 (33.6%)
7–12 mo	14,474 (31.4%)
13–23 mo	14,890 (32.3%)
More than 24 mo	18,885 (41%)
ER/hospitalization due to pancreatic-related conditions within 1 y before index scan	3927 (8.5%)
Statin use	
Within 6 mo	22,313 (48.5%)
7–12 mo	21,478 (46.6%)
13–23 mo	22,629 (49.1%)
More than 24 mo	25,417 (55.2%)
Metformin use	
Within 6 mo	7261 (15.8%)
7–12 mo	7087 (15.4%)
13–23 mo	7699 (16.7%)
More than 24 mo	10,128 (22.0%)
Laboratory measures on t_0_ or in 1 y before t0, median (IQR)	
Alkaline phosphatase (ALP), n = 32,750	74 (59.0, 99.0)
Alanine transaminase (ALT), n = 41,300	22 (16.0, 32.0)
Total bilirubin, n = 32,825	0.7 (0.5, 1.1)
Blood urea nitrogen (BUN), n = 35,694	15 (11.0, 21.0)
Calcium, n = 23,383	9.1 (8.7, 9.5)
Creatinine, n = 45,289	0. 9 (0.8, 1.1)
Hematocrit (HCT), n = 43,332	39.3 (35.6, 42.5)
Hemoglobin (HGB), n = 43,306	13.2 (11.8, 14.3)
Lipase, n = 23,005	29 (21.0, 44.0)
Platelets, n = 43,312	229 (183.0, 283.0)
Red blood cell (RBC), n = 43,133	4.4 (3.9, 4.7)
Sodium, n = 42,690	138 (136.0, 140.0)
Albumin, n = 18,953	3.4 (2.9, 3.8)
High-density lipoproteins (HDL), n = 32,996	48 (40.0, 59.0)
Low-density lipoproteins (LDL), n = 32,669	90 (69.0, 116.0)
Total cholesterol, n = 33,131	167 (139.0, 198.0)
Triglycerides, n = 32,043	116 (84.0, 165.0)
Glycated hemoglobin (HgbA_1c_), n = 28,576	6.2 (5.7, 7.1)
Laboratory change within 1-y before t_0_, median (IQR)	
ALT, n = 20,523	0.0 (−4.0, 6.0)
Total bilirubin, n = 9227	0.1 (−0.2, 0.3)
Bun, n = 12,813	0.0 (−4.0, 4.0)
Creatinine, n = 28,639	0.0 (−0.1, 0.1)
HCT, n = 23,579	−0.7 (−3.1, 1.4)
Hemoglobin, n = 23,568	−0.2 (−1.0, 0.4)
Platelets, n = 23,156	−3.0 (−28.0, 22.0)
RBC, n = 22,992	−0.1 (−0.3, 0.1)
Sodium, n = 23,075	−1.0 (−3.0, 1.0)
HDL, n = 13,132	0.0 (−5.0, 4.0)
LDL, n = 13,097	−3.0 (−18.0, 11.0)
Total cholesterol, n = 13,187	−4.0 (−23.0, 13.0)
Triglycerides, n = 12,329	−2.0 (−32.0, 25.0)
HgbA_1c_, n = 15,069	0.0 (−0.4, 0.3)
Symptoms before the t_0_	
Abdominal pain	
Within 6 mo	15,448 (33.6%)
7–12 mo	5979 (13.0%)
13–23 mo	8385 (18.2%)
More than 24 mo	20,703 (45.0%)
Anorexia	
Within 6 mo	440 (1.0%)
7–12 mo	171 (0.4%)
13–23 mo	228 (0.5%)
More than 24 mo	537 (1.2%)
Back pain	
Within 6 mo	9829 (21.3%)
7–12 mo	8210 (17.8%)
13–23 mo	11,824 (25.7%)
More than 24 mo	24,473 (53.2%)
Chest pain	
Within 6 mo	3674 (8.0%)
7–12 mo	2900 (6.3%)
13–23 mo	4718 (10.2%)
More than 24 mo	17,228 (37.4%)
Constipation	
Within 6 mo	5017 (10.9%)
7–12 mo	2888 (6.3%)
13–23 mo	4223 (9.2%)
More than 24 mo	11,111 (24.1%)
Diarrhea	
Within 6 mo	4124 (9.0%)
7–12 mo	2329 (5.1%)
13–23 mo	3480 (7.6%)
More than 24 mo	11,250 (24.4%)
Itching	
Within 6 mo	2199 (4.8%)
7–12 mo	1922 (4.2%)
13–23 mo	3221 (7.0%)
More than 24 mo	10,397 (22.6%)
Malaise/fatigue	
Within 6 mo	8056 (17.5%)
7–12 mo	5643 (12.3%)
13–23 mo	8255 (17.9%)
More than 24 mo	19,030 (41.3%)
Melena	
Within 6 mo	979 (2.1%)
7–12 mo	525 (1.1%)
13–23 mo	858 (1.9%)
More than 24 mo	3977 (8.6%)
Nausea or vomiting	
Within 6 mo	6592 (14.3%)
7–12 mo	3186 (6.9%)
13–23 mo	4579 (9.9%)
More than 24 mo	12,467 (27.1%)
Weight loss	
Within 6 mo	4110 (8.9%)
7–12 mo	1783 (3.9%)
13–23 mo	2572 (5.6%)
More than 24 mo	7398 (16.1%)
GERD	
Within 6 mo	7381 (16.0%)
7–12 mo	5999 (13.0%)
13–23 mo	8216 (17.8%)
More than 24 mo	16,814 (36.5%)
Abdominal bloating	
Within 6 mo	1230 (2.7%)
7–12 mo	618 (1.3%)
13–23 mo	850 (1.8%)
More than 24 mo	3625 (7.9%)
Dyspepsia	
Within 6 mo	1415 (3.1%)
7–12 mo	829 (1.8%)
13–23 mo	1365 (3.0%)
More than 24 mo	7192 (15.6%)
Dysphagia	
Within 6 mo	865 (1.9%)
7–12 mo	606 (1.3%)
13–23 mo	906 (2.0%)
More than 24 mo	3351 (7.3%)

### Imaging Features

For each patient, we defined the presence/absence of each feature (duct dilatation, atrophy, calcification, cyst, or pseudocyst) using NLP based on the index scans and all the abdominal scans available in the KPSC system between January 1, 2004, and t_0_. The NLP algorithms to extract the 5 features were previously described.^11^

### Model Training and Validation Based on Machine Learning

A machine learning method, random survival forests (RSF),^15^, ^16^, ^17^ was used to preselect features and train/validate risk prediction models. The learning process of RSF involves randomly drawn bootstrap samples to be used to grow trees and randomly selected predictors to split nodes. The results are averaged among trees. Compared with the Cox proportional hazards regression model, RSF has the advantages of handling nonlinear effects and interactions among predictors and without needing to test the proportionality assumption.

#### Feature Selection

For each of the 10 imputed data sets, we ran RSF to preselect the most influential features. Those with an average minimum depth of <6.5 (first round) and 5.4 (second round) were identified. To avoid overfitting, we applied 5-fold cross-validation method.^18^ We randomly divided each imputed data set into 5 folds and use the first 4 folds of data for model development and the remaining one fold for validation. Repeat the process 4 more times until each of the 5 folds is left out once for validation.

Based on the preselected features, the following steps were repeated 5 times for each of the 10 imputed data sets to select the most important features.

1. Preselected features that were not in the model were added, one at a time. Each time, the feature that yielded the maximum improvement of c-index was selected.
2. This iterative process continued until the increase of c-index is <0.005.

#### Hyperparameter Setup

The number of trees and depth of trees were set to 100 and 7, respectively. The number of covariates available for splitting at each node (termed “mtry”) was set to be an integer that is close to the square root of the number of covariates.

#### Model Selection

Of the 50 models derived from the 50 training data sets, the ones that appeared the most often were selected as the final models.

#### Model Validation

The algorithms of the winning models were applied to the corresponding validation data sets that were left out for validation. By design, the validation data sets did not include any observations of the training data sets from which the winning models were developed.

#### Performance Measures

The discriminative power for each of the winning models was evaluated by c-index, a concordance measure, pooled across all the relevant validation data sets for cohort members using Rubin's rule implemented in mi.meld function within the R package Amelia.^19^, ^20^, ^21^

Calibration was assessed by calibration plots with 5 risk groups (<50th, 50–74th, 75–89th, 90–94th, and 95–100th percentiles).^22^ The calibration plot was produced for the best model.

### Statistical Analysis

Patient demographic, clinical, and imaging features are reported as n (%), mean (standard deviation), or median (interquartile range) as appropriate. Kaplan-Meier plot was generated to present PDAC-free survival in patients with the presence of one or more imaging features. Overall and risk factor–stratified crude event rates were calculated using log-linear (Poisson) regression with a generalized estimating equations approach and are reported as per 1000 person-years of follow-up. To estimate the effect of the covariates on the risk of PDAC, multivariable Cox proportional hazard model was applied, and hazard ratios (HRs) were reported with 95% confidence intervals (CIs). All the continuous variables were normalized based on z-score standardization before they were applied to the Cox model. To estimate the pooled HR, we combined the HR derived from each of the 10 imputed data sets using Rubin's rule implemented in PROC MIANALYZE in SAS. All the analyses were performed using SAS (Version 9.4 for Unix; SAS Institute, Cary, NC) except for the R packages mentioned previously. All computations and analyses carried out in R were based on R Version 3.6.0 (R Foundation, Vienna, Austria).

## Results

### Characteristics of the Study Cohort

A total of 46,041 patients/examinations met the eligibility criteria (Figure A2; mean age 66.4 years, 55.8% female, 51.2% non-Hispanic White, 27.2% Hispanic, 11.2% African American, and 9.2% Asian and Pacific Islanders), with an average follow-up time of 1.9 years. Patient characteristics are presented in Table 1. Overall, 48.5% of patients were current or ever smokers. Alcohol abuse was reported in 6.8% in the past year and in 11.5% any time in the past. More than 3% had a family history of pancreatic cancer. One-third of study subjects had diabetes, 23.9% had gallstone disorders, and 28.9% had biliary tract disease order. In addition, 4.1% of patients had CP, and 12.6% had acute pancreatitis. The percentage of patients who were hospitalized in the past 12 months for pancreatic-related conditions was 8.5%. The median HbA1c was 6.2 (IQR: 5.7, 7.1). The 2 most common gastrointestinal symptoms were abdominal pain and back pain (33.6% and 21.3% in the 6 months before the index scan, respectively).

In terms of the imaging findings, 6753 (14.7%) patients were identified based on MRI, and 39,288 (85.3%) were identified based on CT scan (Table 2). A majority (77.8%) were performed in an outpatient or emergency department setting. Atrophy (31.2%) and cyst (31.8%) were the most common imaging abnormalities, followed by calcification (27.4%) and duct dilatation (22.6%). Overall, 17.4% of patients had more than one abnormal morphologic feature. Abdominal pain was the most common indication for the index scan accounting for 25.2% of study subjects. Other common indicators included gastrointestinal problem (13.1%), other pain (10.9%), and concern raised by laboratory test results (9.9%).

**Table 2:** Imaging-Related Characteristics of Study Subjects Presented on or Before t_0_, Extracted by Natural Language Processing (NLP; n = 46,041)

Patient characters related to image	n (%)
Imaging features (mutually inclusive) at or before index scan:	
Atrophy	14,343 (31.2)
Calcification	12,637 (27.4)
Cyst	14,661 (31.8)
Duct dilatation	10,413 (22.6)
Pseudocyst	4435 (9.6)
Imaging features (mutually exclusive) at or before index scan:	
Single	
Calcification only	9437 (20.5)
Duct dilatation only	6667 (14.5)
Atrophy only	11,026 (23.9)
Cyst only	9269 (20.1)
Pseudocyst only	1636 (3.6)
Two or more	
Calcification + duct dilatation (w/ or wo/ atrophy, cyst, pseudocyst)	1197 (2.6)
Calcification + any 1 or more of (atrophy, cyst, pseudocyst)	2003 (4.4)
Duct dilatation + any 1 or more of (atrophy, cyst, pseudocyst)	2549 (5.5)
Any 2 or more of (atrophy, cyst, pseudocyst)	2257 (4.9)
Type of service at index scan	
Outpatient/ED	35,802 (77.8)
Inpatient	10,239 (22.2)
Index scan modality	
CT	39,288 (85.3)
MRI	6753 (14.7)
Indication for the index scan (mutually inclusive)	
Abdominal pain	11,622 (25.2)
Other pain	5037 (10.9)
GI problem	6022 (13.1)
Concern raised by laboratory test results	4568 (9.9)
Follow-up	3661 (8.0)
Urinary problem	2323 (5.1)
Consultation	2055 (4.5)
Shortness of breath	950 (2.1)
Weakness	930 (2.0)
Fever	749 (1.6)
Nonpancreatic cancer	453 (1.0)
Others	9496 (20.6)
Unknown	9108 (19.8)

### Incidence of PDAC

Of 46,041 eligible patients, 370 developed PDAC within 3 years with an incidence rate of 4.0/1000 (95% CI 3.6–4.4/1000) person-years of follow-up. The median follow-up time for PDAC cases was 96 days (interquartile range, 49–294 days). Of the 370 PDAC cases, 296 (80%) were captured from the KPSC Cancer Registry, and the rest (74 or 20%) died of pancreatic cancer based on the information with the CA State death files. The total follow-up time in years, mean follow-up time per patient, number (and incidence rate) of PDAC, and time to PDAC diagnosis or death are reported in Table 3. In terms of individual findings, main duct dilatation was associated with the highest incidence of PDAC (Table 3).

**Table 3:** Total and Per Patient Follow-Up (f/u) Time, Number, and Incidence Rate of PDAC per 1000 Person-Years (PY) and 95% Confidence Interval (CI)

Patient characteristics	Total f/u time (y)	Average f/u time (y)	No. of PDAC events	Incidence rate of PDAC/1000 PY (95% CI)	Time to PDAC (days) (median, IQR)
All	88,550	1.9	370	4.2 (3.8, 4.6)	96 (49, 294)
Age group					
18–49	10,491	2.0	13	1.2 (0.7, 2.1)	56 (40, 64)
50–59	15,124	2.1	49	3.2 (2.4, 4.3)	75 (46, 240)
60–69	24,610	2.0	102	4.1 (3.4, 5.0)	83 (45, 235)
70–79	28,882	2.0	153	5.3 (4.5, 6.2)	127 (57, 348)
80–84	9443	1.4	53	5.6 (4.3, 7.4)	123 (45, 325)
Sex					
Female	50,083	1.9	168	3.4 (2.9, 3.9)	103 (57, 319)
Male	38,467	1.9	202	5.3 (4.6, 6.0)	93 (42, 267)
Race/ethnicity					
Non-Hispanic White	45,531	1.9	182	4.0 (3.5, 4.6)	121 (52, 295)
African American	10,134	2.0	60	5.9 (4.6, 7.6)	82 (47, 141)
Hispanic	23,639	1.9	93	3.9 (3.2, 4.8)	107 (49, 403)
Asian/Pacific Islanders	8303	2.0	34	4.1 (2.9, 5.7)	65 (40, 118)
Unknown	943	1.7	1	1.1 (0.1, 7.5)	142 (142, 142)
Weight change in 1 y (kg)					
≤−6 kg	11,806	1.7	118	10.0 (8.3, 12.0)	89 (44, 267)
>−6 and ≤−4 kg	6989	1.9	43	6.2 (4.6, 8.3)	78 (37, 232)
>−4 and ≤−2 kg	10,588	1.9	43	4.1 (3.0, 5.5)	141 (66, 454)
>−2 and <2 kg	28,663	2.0	85	3.0 (2.4, 3.7)	103 (57, 274)
≥2 and <4 kg	7515	2.0	17	2.3 (1.4, 3.6)	132 (67, 308)
≥4 kg	8406	1.9	16	1.9 (1.2, 3.1)	122 (68, 559)
Unknown	14,582	2.0	48	3.3 (2.5, 4.4)	67.5 (46, 137)
Family history of pancreatic cancer					
No	85,761	1.9	339	4.0 (3.6, 4.4)	94 (48, 286)
Yes	2789	1.9	31	11.1 (7.8, 15.8)	150 (53, 336)
Imaging features					
Single					
Calcification only	19,363	2.1	22	1.1 (0.7, 1.7)	131 (56, 412)
Duct dilatation only	13,307	2.0	110	8.3 (6.8, 10.0)	74 (42, 166)
Atrophy only	19,711	1.8	32	1.6 (1.1, 2.3)	249 (61, 518)
Cyst only	18,377	2.0	41	2.2 (1.6, 3.0)	120 (54, 333)
Pseudocyst only	3315	2.0	10	3.0 (1.6, 5.6)	117 (62, 229)
Two or more					
Calcification + duct dilatation (w/ or wo/ atrophy, cyst, pseudocyst)	2193	1.8	27	12.3 (8.4, 18.0)	99 (44, 454)
Calcification + any 1 or more of (atrophy, cyst or pseudocyst)	3695	1.8	8	2.2 (1.1, 4.3)	297 (125, 519)
Duct dilatation + any 1 or more of (atrophy, cyst or pseudocyst)	3952	1.6	99	25.1 (20.5, 30.6)	78 (43, 204)
Any 2 or more of (atrophy, cyst, pseudocyst)	4636	2.1	21	4.5 (3.0, 7.0)	294 (69, 530)
ALP values at baseline					
≤125	53,696	1.9	193	3.6 (3.1, 4.1)	118 (56, 307)
>125	7610	1.7	117	15.4 (12.8, 18.5)	68 (43, 145)
Unknown	27,244	2.0	60	2.2 (1.7, 2.8)	120 (46, 446)
Lipase values at baseline					
<60	35,442	1.8	139	3.9 (3.3, 4.6)	102 (49, 333)
[60, 180]	4422	1.8	48	10.9 (8.2, 14.4)	69 (41, 250)
≥180	2561	1.9	55	21.5 (16.4, 28.1)	91 (52, 178)
Unknown	46,125	2.0	128	2.8 (2.3, 3.3)	112 (49, 345)
Total bilirubin values at baseline					
≤1	46,501	1.9	179	3.8 (3.3, 4.5)	111 (56, 279)
>1	14,901	1.8	129	8.7 (7.3, 10.3)	71 (44, 232)
Unknown	27,148	2.1	62	2.3 (1.8, 2.9)	103 (44, 438)
HbA1c values at baseline					
<6.5%	30,601	1.8	113	3.7 (3.1, 4.4)	107 (52, 342)
6.5%–6.9%	6745	1.9	33	4.9 (3.5, 6.9)	76 (41, 200)
7%–7.4%	4680	1.9	26	5.6 (3.8, 8.2)	79 (43, 294)
≥7.5%	9956	1.8	78	7.8 (6.3, 9.8)	103(54, 317)
Unknown	36,567	2.1	120	3.3 (2.7, 3.9)	97 (48.5, 269.5)
ALT change within 1 y prior					
<−5	8322	1.9	28	3.4 (2.3, 4.9)	147 (66, 344)
[−5, 5]	20,194	2.0	69	3.4 (2.7, 4.3)	146 (54, 357)
≥5	11,351	1.9	78	6.9 (5.5, 8.6)	73 (40, 182)
Unknown	48,683	1.9	195	4.0 (3.5, 4.6)	94 (52, 295)
Abdominal pain within 6 mo					
No	58,503	1.9	195	3.3 (2.9, 3.8)	143 (57, 403)
Yes	30,047	1.9	175	5.8 (5.0, 6.8)	75 (43, 190)
Dyspepsia within 6 mo					
No	85,695	1.9	345	4.0 (3.6, 4.5)	102 (49, 300)
Yes	2855	2.0	25	8.8 (5.9, 13.0)	59 (44, 163)
Malaise/fatigue					
No	40,301	2.0	186	4.6 (4.0, 5.3)	87 (48, 294)
Yes	48,249	1.8	184	3.8 (3.3, 4.4)	103 (50, 290)
Weight loss					
No	66,058	2.0	244	3.7 (3.3, 4.2)	93 (52, 285)
Yes	22,492	1.8	126	5.6 (4.7, 6.7)	110 (45, 307)

ALP, alkaline phosphatase; ALT, Alanine transaminase.

**Figure 1:**
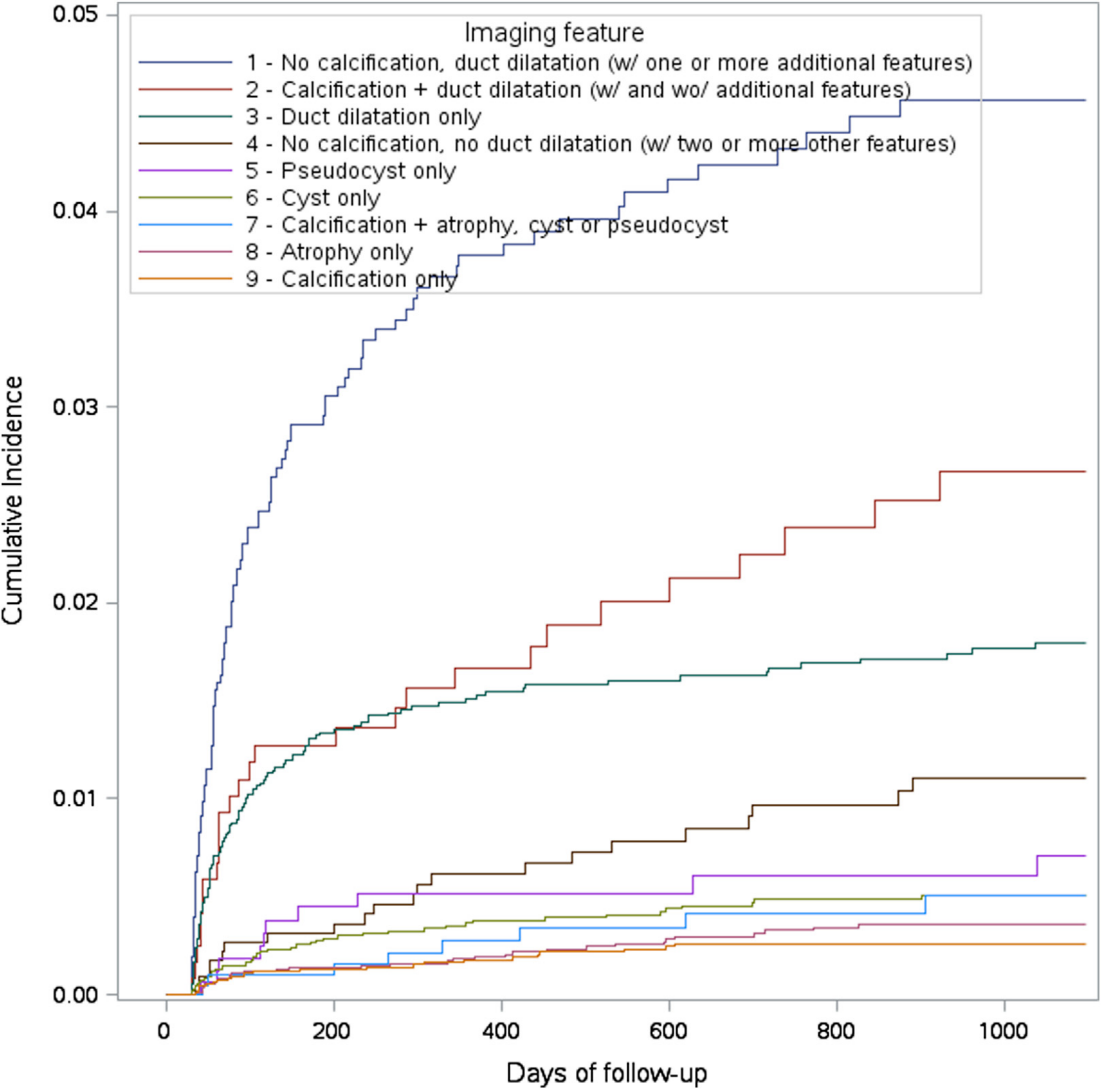
The cumulative incidence of PDAC in 3 years by imaging feature. The order of the descriptions in the legend and the order of the curves match.

The cumulative incidence of PDAC in 3 years by imaging feature is displayed in Figure 1. The observed incidence of PDAC was further elevated among patients, with main duct dilatation combined with additional findings, particularly in the absence of calcification (Table 3). Patients without calcification but with pancreas duct dilatation and one or more other feature(s) had the highest incidence rate, followed by patients with both calcification and pancreas duct dilatation and patients with only duct dilatation (Table 3, Figure 1).

Among the patients whose cancer stage was known (n = 210), 37 (17.6%), 93 (44.3%), 20 (9.5%), and 60 (28.6%) had stage I, stage II, stage III, and stage IV cancer, respectively.

### Demographic and Clinical Parameters Associated With Increased Risk of PDAC

In addition to imaging-based risk, various demographic and clinical parameters were associated with increased risk of PDAC (Table 3). Older age, male sex, and African American race were each associated with higher risk of cancer. Family history of PDAC was also associated with increased risk. In terms of clinical parameters weight loss in the past year, elevated alkaline phosphatase (ALP), lipase, bilirubin, or glycated hemoglobin value at the time of index scan was associated with increased PDAC incidence. In addition, increased extent of alanine transaminase change within the past 1 year was associated with a higher PDAC incidence.

### Risk Factors Associated With the Risk of PDAC Based on Cox Regression Analysis

The adjusted HRs and 95% CIs for risk of PDAC from a multivariable model incorporating the aforementioned risk factors for PDAC are reported in Table 4. In terms of imaging findings, patients with pancreas duct dilatation and at least another morphological feature in the absence of calcification had the highest risk of developing PDAC (aHR = 14.15, 95% CI 8.7–22.6), followed by patients with calcification and duct dilatation (aHR = 7.28, 95% CI 4.09–12.96), patients with duct dilation only (aHR = 6.22, 95% CI 3.86–10.03), patients with 2 or more features of atrophy, cyst, or pseudocyst (aHR = 3.77, 95% CI 2.04–6.95), and patients with cyst or pseudocyst only (aHR = 2.26, 95% CI 1.36–3.75), compared with patients with calcifications alone as the reference group. Other risk factors and their estimated effects are listed in Table 4.

**Table 4:** Adjusted Hazard Ratio and 95% Confidence Interval (CI) of 3-y PDAC

Patient characteristics	HR	95% CL	
		LL	UL
Imaging feature (ref = Calcification only)			
Single			
Duct dilatation only	6.22	3.86	10.03
Atrophy only	1.21	0.70	2.11
Cyst only or pseudocyst only	2.26	1.36	3.75
Two or more			
Calcification + duct dilatation (w/ or wo/ atrophy, cyst, pseudocyst)	7.28	4.09	12.96
Calcification + any 1 or more of (atrophy, cyst, pseudocyst)	1.58	0.70	3.55
Duct dilatation + any 1 or more of (atrophy, cyst, pseudocyst)	14.05	8.71	22.64
Any 2 or more of (atrophy, cyst, pseudocyst)	3.77	2.04	6.95
Index scan setting (inpatient vs outpatient; ref = outpatient)	0.69	0.52	0.92
Age	1.51	1.30	1.76
Age^2^	0.77	0.67	0.90
Male (ref = female)	1.62	1.29	2.02
Family history of PC	2.64	1.82	3.83
Weight loss (every 10 kg)	1.68	1.48	1.91
ALP (unit of increase 1 SD)	1.12	1.05	1.18
HbA1c (unit of increase 1 SD)	1.34	1.24	1.45
Lipase (unit of increase 1 SD)	1.17	1.10	1.24
Total bilirubin (unit of increase 1 SD)	1.17	1.11	1.23
Change in ALT (unit of increase 1 SD)	1.11	1.02	1.21
Abdominal pain within 6 mo	1.87	1.51	2.32
Dyspepsia within 6 mo	1.56	1.03	2.38
Malaise/fatigue within 6 mo	0.73	0.55	0.98

ALP, alkaline phosphatase; ALT, alanine transaminase; CL, confidence limit; HR, hazard ratio; LL, lower limit; UL, upper limit.

### Risk Prediction Models Based on RSF Analysis

The preselection process identified 14–21 potential predictors from the 10 imputed data sets. Of the 50 training data sets, the models with age, weight change, duct dilatation, and either ALP or total bilirubin appeared most often (Table 5). A summary of training and validation data sets can be found in Table A2 of the Online Document.

**Table 5:** Frequency of the Selected Models Based on the 50 Training Data Sets and the Average Performance Measured by c-Index of These Models Based on the Holdout Validation Data Sets

Models formed	No. of times selected out of 50 training samples	Mean c-index (SD) based on 50 validation datasets
Age, weight change, duct dilatation, ALP	4	0.811 (0.037)
Age, weight change, duct dilatation, total bilirubin	3	0.805 (0.013)

ALP, alkaline phosphatase.

The mean and standard deviation of c-index based on the validation data sets for each winning model are reported in Table 5. The c-indices were high for both models (0.811 for the model with ALP and 0.805 for the model with total bilirubin). The calibration plot based on age, weight change, duct dilatation, and ALP was displayed in Figure A2. The differences between the average predicted and averaged observed differences were small for the 3 lowest risk groups (Figure A2). Although the differences appeared to be somewhat large in the 2 highest risk groups (Figure A2), the ranges of the absolute difference between the predicted and the observed were only 0.07%–0.22% (data not shown). The calibration plot for the model with bilirubin was similar (data not shown).

## Discussion

In this study, we performed a comprehensive assessment of the relationship between common parenchymal and ductal abnormalities of the pancreas on cross-sectional imaging with the risk of pancreatic cancer. Specifically, we applied NLP to identify a large cohort of patients with the presence of at least one feature commonly associated with CP: main duct dilatation, atrophy, cyst/pseudocyst, or calcification. The implementation of NLP makes the information extraction feasible for a large cohort of patients. We then performed traditional Cox regression analysis to assess the relative risk of developing PDAC based on individual as well combinations of imaging findings in addition to patient demographic and clinical parameters. Finally, we developed and validated risk prediction models using an empiric machine learning–based approach (RSF) to optimize the use of patient demographic, clinical, as well as imaging data for the prediction of 3-year risk of PDAC. The final models were able to achieve a high level of discrimination (c-index of 0.81) with acceptable calibration (absolute risk difference predicted vs predicted 0.07%–0.22%) for 3-year risk of PDAC.

Of the 5 morphological features we studied, the associations between main duct dilatation,^23^, ^24^, ^25^, ^26^ pancreatic parenchymal atrophy,^27^, ^28^, ^29^ chronic calcific pancreatitis,^30^^,^^31^ and pancreatic cyst^26^^,^^32^, ^33^, ^34^ with pancreatic cancer have been previously reported in smaller case–control studies. In the present study, we developed and validated risk prediction models based on these morphological features using a much larger data set, including additional patient demographic and clinical features. We also reported the absolute risks and the relative risks of the individual morphological features.

Pancreatic cancer is a devastating disease and represents the third leading cause of cancer-related death among cancers that afflict men and women in the United States.^1^ A major factor contributing to the lethal nature of PDAC is the advanced stage at presentation, with more than 50% of patients having distant metastases at the time of diagnosis.^2^^,^^3^ Therefore, approaches for early detection are urgently needed to improve patient outcomes. However, due in part to the relatively rare nature of PDAC (incidence 14 in 100,000), the United States Preventative Services Task Force recently reissued guidance against widespread population-based screening for PDAC.^4^ Another key barrier to early detection in PDAC has also been the inability to identify precursor lesions on conventional imaging.

We hypothesized that changes related to early cancer-related desmoplasia might be visible on cross-sectional imaging and could share the appearance of features typically associated with CP. A hallmark of PDAC is a dense surrounding stromal response consisting of extracellular matrix proteins, activated myofibroblasts (stellate cells), and inflammatory cells described as desmoplasia.^7^ This stroma can constitute up to 90% of tumor volume.^35^ The tumor microenvironment also plays a key role in early tumor progression.^36^, ^37^, ^38^, ^39^ Although the precursor lesion to PDAC, Pancreatic Intraepithelial Neoplasia type III (PanIN III) or high-grade dysplasia,^40^ is a microscopic lesion that is not visible on cross-sectional imaging, it is conceivable that changes in pancreas morphology related to early cancer-related desmoplasia can be identified before tumor diagnosis. In particular, we assessed features commonly associated with CP, given shared mechanistic pathways with activated pancreatic stellate cells playing a key role in mediating extracellular matrix deposition.^41^ Our hypothesis was supported by the low proportion of patients with a clinical diagnosis of either acute or CP in the imaging-based study cohort, 12.6% and 4.1%, respectively, as well as the relatively short interval to cancer diagnosis (median 96 days).

**Figure 2:**
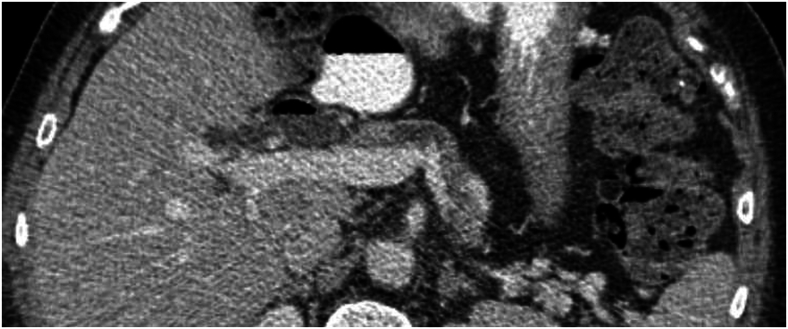
Pancreas with duct dilatation and atrophy involving the body as well as tail of pancreas 2 years before cancer diagnosis.

Understanding the relationship between individual and combinations of imaging findings with the risk of PDAC can help develop a profile for imaging changes during early cancer development. Of the 5 morphological features included in the study, pancreas duct dilatation, either alone or in combination with one or more other morphological features, significantly increased the risk of PDAC. This finding is consistent with previous studies associating early findings of pancreas duct dilatation with the development of pancreatic cancer.^23^^,^^24^ In the study of Singh et al,^24^ abrupt pancreas duct cut-off/duct dilatation were seen on CT images 12.8 months before cancer diagnosis. A review of Gangi et al^23^ revealed that definite or suspicious findings (predominantly duct dilatation) based on CT studies were present in 50% of the CTs obtained in the 6–18 months before the diagnosis of pancreatic cancer. However, the median time to cancer diagnosis among patients with duct dilatation was only 74 days, indicating this is likely a very late event in tumor development. In contrast, other findings such as parenchymal atrophy were associated with a longer interval before cancer diagnosis. This observation combined with that of patients with duct dilatation in conjunction with other imaging abnormalities conferred greatest risk, and most rapid onset of PDAC argues for a sequential accumulation of imaging findings potentially corresponding with stages of early tumorigenesis as illustrated in Figure 2.

As the imaging findings included in the present study can also be seen in the setting of age-related changes or conditions other than PDAC, we set about determining additional clinical parameters that would enhance specificity for early cancer-related morphologic changes. In addition to established risk factors such as advancing age and family history,^42^ weight loss, and elevated A1c,^43^^,^^44^ elevation in lipase level and alterations in liver tests were also associated with the development of PDAC. Among these clinical parameters, weight loss was associated with the longest interval to cancer diagnosis consistent with previous studies among patients with new-onset diabetes.^45^ Weight loss in the setting of one of the aforementioned imaging abnormalities would raise suspicion for cancer-related changes. This also supports previous observations that cancer-related cachexia in PDAC can begin before tumor diagnosis potentially mediated by alterations in body fat composition.^46^^,^^47^

The empiric machine learning–based prediction models were developed to enhance the specify of imaging findings for the identification of cancer-related changes as well as demonstrate the potential accuracy of combining data from imaging reports with clinical parameters from the EHR. The final models selected by the algorithm were parsimonious, containing only 4 parameters: age, duct dilatation, weight loss, and a measure of cholestasis (ALP or bilirubin). These models could have several future applications in terms of research including integration with emerging blood-based biomarkers for early detection of PDAC. In addition, such a model could be included as an automated algorithm for enhanced radiology reporting of PDAC risk when pancreatic abnormalities are identified in the context of routine clinical care.

Although malaise/fatigue is a known risk factor of PDAC, it was found to be a protective factor in the present study. This could be at least partially attributed to non-PDAC cancers, which also causes malaise and fatigue. Overall, 7.6% of the study subjects had active cancer other than PDAC, and the risk of PDAC in this group of patients is lower.

There were several limitations in the present study. First, the images used for analysis were acquired in the context of routine clinical care, and as a result, there was variation in types of studies and imaging protocols used. This may have caused inconsistency in the interpretation of the imaging reports. Second, the study population was heterogeneous with respect to the indications for imaging. It is therefore unclear how the present study findings would extend to an asymptomatic population undergoing screening. However, the findings do reflect conditions in real-world practice. Third, it is possible that some of the desired features may not have been reported by radiologists as part of a clinical reading for a nonpancreas-related indication. Thus, the prevalence of the abnormalities may be higher than what was reported. A direct imaging analysis in the future to extract pancreas morphological features could minimize the issue.

Also, the current analysis looked only at morphologic imaging features on CT and MRI. The analysis did not include evaluation of newer oncologic imaging techniques in MRI, such as diffusion weighted imaging, or quantitative measures such as differential contrast enhancement on both single and dual-energy CT (delta). Studies have shown diffusion weighted imaging is helpful in distinguishing pancreatic cancer from acute or CP.^48^ Differential contrast enhancement (high delta) has been shown to aid in the identification of pancreatic neoplasms^49^ as well as correlate with prognosis.^50^ It is possible that some of these features could be even more predictive, and assessment of other features provides opportunity for future research.

Despite the aforementioned limitations, the present study has some key strengths that have enabled us to glean new insights into the relationship between specific imaging findings and early pancreatic cancer. First, by scaling up a previously developed automated natural language algorithm for pancreas findings on the free text of radiology reports, we were able to identify a large cohort of patients with the features of interest on cross-sectional imaging. By combining this approach with comprehensive data from a robust electronic health system within an integrated care system, we were able to reliably ascertain both patient-related clinical characteristics as well as robust ascertainment of cancer diagnoses. Finally, by incorporating state-of-the-art machine learning approaches to predictive modeling, we were able to achieve a high degree of accuracy for discrimination of findings suggestive of early cancer by combining structured data from the EHRs as well as unstructured data from radiology reports acquired in the context of routine clinical care.

In conclusion, we have characterized the risk of pancreatic cancer among patients with 5 abnormal morphologic findings based on radiology reports and demonstrated the ability of prediction algorithms to provide improved risk stratification of pancreatic cancer in these patients. We have further mapped the temporal development of imaging abnormalities in relation to cancer diagnosis, which suggests an accumulation of derangements that may parallel early tumorigenesis with main duct dilatation representing one of the last developments in this sequence. Based on our initial hypothesis, the overlap of morphologic changes seen before PDAC diagnosis with classic features of CP likely represents macroscopic changes associated with the stromal response in early tumorigenesis seen in PDAC rather than the tumor itself. Although much additional investigation is needed, these findings suggest that features associated with cancer-related desmoplasia may be visualized before cancer development and therefore provide a suitable target for early detection as well as provide a critical window for potential intervention or perhaps even prevention by applying therapy directed at altering the tumor microenvironment before frank tumor development.

## Supplementary Materials

Tables A1 and A2

Figure A1 Consort diagram for cohort identification

Figure A2 Calibration plots of the mode based on age, weight change, duct dilatation, ALP was displayed in Figure. x-axis: predicted; y-axis: observed. The 5 clusters represent the 5 risk groups defined by the ranges of predicted risks: <50th, 50–74th, 75–89th, 90–94th, and 95–100th percentiles. Within each cluster, there are multiple dots representing the pairs of predicted and observed risks, calculated based on the corresponding validation data sets
