# Supplementary material for: Risk Prediction of Pancreatic Cancer in Patients With Abnormal Morphologic Findings Related to Chronic Pancreatitis: A Machine Learning Approach
Source: Gastro Hep Adv. 2022 Jun 17;1(6):1014–26. doi: 10.1016/j.gastha.2022.06.008 (PMC9718544; doi:10.1016/j.gastha.2022.06.008)
Supplement: Tables A1 and A2 [file mmc1.docx]

| **Datasets** | **Number** | **Size** | **Proportion who censored** | **Proportion with PDAC in 3 years** | **Proportion who died** |
| --- | --- | --- | --- | --- | --- |
| Training | 50 | 36832-36833 | 87.58-87.92 | 0.75-0.85 | 11.31-11.60 |
| Validation | 50 | 9208-9209 | 87.15-88.52 | 0.61-1.02 | 10.72-11.89 |

**Online document**

**eTable 1. Summary of the training, and validation datasets.**

**eTable 2. ICD-9 and ICD-10 codes used to define medical conditions.**

|  | **ICD-9** | **ICD-10** |
| --- | --- | --- |
| **Alcohol abuse** | 291.X 303.0 303.9 305.0 357.5 425.5 535.3 571.0 571.2 571.3 760.71 980.0 980.1 E860.1 E860.2 E860.9 | K70.X F10.0 F10.1F F10.2 F10.9 G62.1 G31.2 G72.1 I42.6 K29.2  Q86.0 P04.3 O35.4 K86.0 T51.0 T51.1 T51.9 R78.0 |
| **Gallstone disorders** | 574.X 576.1 | K80.X K83.0 |
| **Acute pancreatitis** | 577 | K85.X |
| **Chronic pancreatitis** | 577.1 | K86.0 K86.1 |
| **Benign pancreatic disease** | 577.2 | K86.2 K86.3 |
| **Biliary tract disease** | 574.X 575.X 576.X 793.3 | K80.X K81.X K82.X K83.X K87.X K91.5 R93.2 |
| **Depression** | 311.X 296.0-296.7 296.80 296.82 296.89 | F30.X F31.X F32.X F33.X |
| **Deep vein thrombosis** | 453.0 453.2 453.3 453.4 453.8 | I82.0 I82.210 I82.220 I82.290 I82.3 T82.4 I82.6 I82.A1 I82.B1 I82.C1 I82.890 I82.90 |
| **Hereditary cancer syndromes*** | V84.X V16.0 V18.51 | D12.2 D12.3 D12.4 D12.6 Z15.0 Z83.71 Z80.0 |
| **Peptic ulcer*** | 531.x 532.X 533.X 534.X | K25.X K26.X K27.X K28.X P78.82 |
| **Active cancer (other than pancreatic cancer)** | With infusion treatment within 1-year prior to index date | |

*Additional KP internal codes were used
