# Supplementary material for: Risk Prediction of Pancreatic Cancer in Patients With Abnormal Morphologic Findings Related to Chronic Pancreatitis: A Machine Learning Approach
Source: Gastro Hep Adv. 2022 Jun 17;1(6):1014–26. doi: 10.1016/j.gastha.2022.06.008 (PMC9718544; doi:10.1016/j.gastha.2022.06.008)
Supplement: Figure A1 — Consort diagram for cohort identification [file mmc2.pdf]

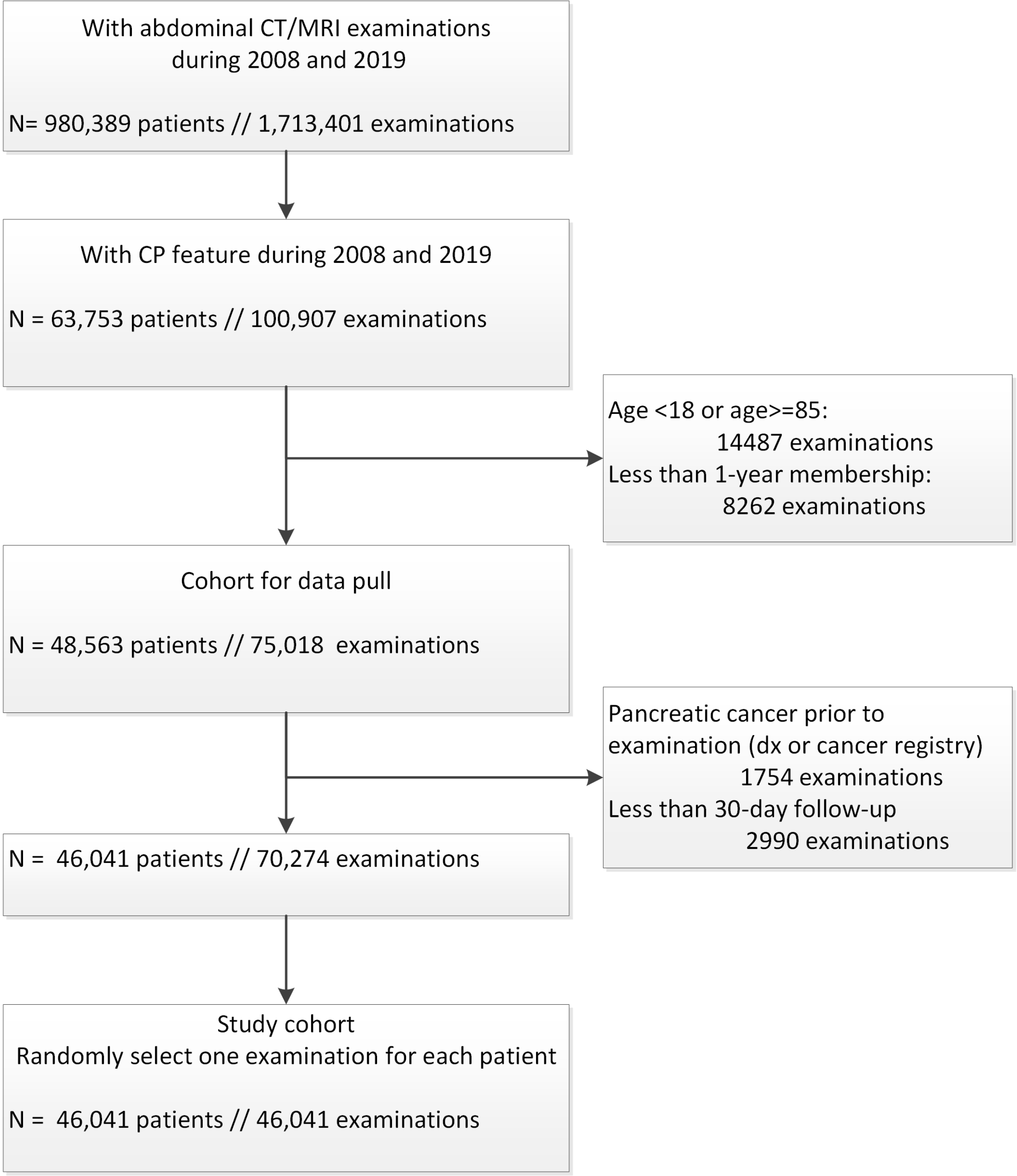
With abdominal CT/MRI examinations during 2008 and 2019
N= 980,389 patients // 1,713,401 examinations
With CP feature during 2008 and 2019
N = 63,753 patients // 100,907 examinations
Age <18 or age>=85: 14487 examinations
Less than 1-year membership: 8262 examinations
Cohort for data pull
N = 48,563 patients // 75,018 examinations
Pancreatic cancer prior to examination (dx or cancer registry) 1754 examinations
Less than 30-day follow-up 2990 examinations
N = 46,041 patients // 70,274 examinations
Study cohort
Randomly select one examination for each patient
N = 46,041 patients // 46,041 examinations
