# Supplementary figures and images for: Risk Prediction of Pancreatic Cancer in Patients With Abnormal Morphologic Findings Related to Chronic Pancreatitis: A Machine Learning Approach
Source: Gastro Hep Adv. 2022 Jun 17;1(6):1014–26. doi: 10.1016/j.gastha.2022.06.008 (PMC9718544; doi:10.1016/j.gastha.2022.06.008)

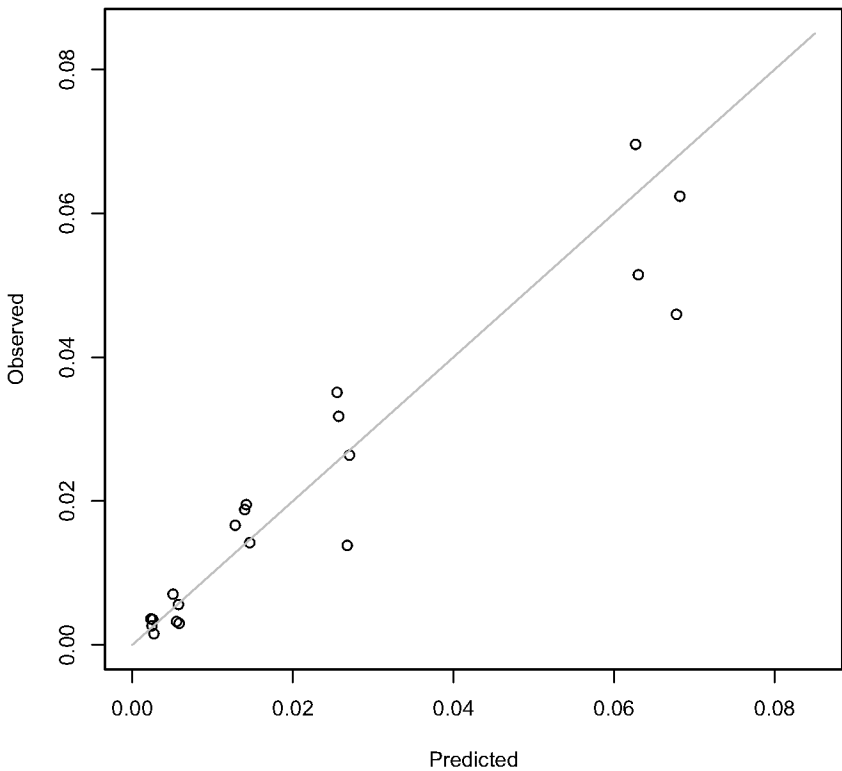

Supplement: Figure A2 — Calibration plots of the mode based on age, weight change, duct dilatation, ALP was displayed in Figure. x-axis: predicted; y-axis: observed. The 5 clusters represent the 5 risk groups defined by the ranges of predicted risks: <50th, 50–74th, 75–89th, 90–94th, and 95–100th percentiles. Within each cluster, there are multiple dots representing the pairs of predicted and observed risks, calculated based on the corresponding validation data sets [file mmc3.pdf]
